# Exclusive Feature Papers in Synthetic Medicinal Chemistry

**DOI:** 10.3390/molecules31142454

**Published:** 2026-07-14

**Authors:** Michael S. Christodoulou, Constantinos M. Athanassopoulos

**Affiliations:** 1 Department of Food, Environmental and Nutritional Sciences (DeFENS), University of Milan, Via Celoria 2, 20133 Milan, Italy; 2 Synthetic Organic Chemistry Lab, Department of Chemistry, University of Patras, GR-26504 Patras, Greece

The collection of manuscripts presented in this issue exemplifies the breadth and vitality of contemporary medicinal chemistry, demonstrating how innovative molecular design, sophisticated synthetic methodologies, and biologically driven investigations continue to expand the frontiers of chemical and pharmaceutical sciences. Although the contributions span diverse research areas—from nucleoside chemistry and heterocyclic anticancer agents to natural product synthesis and comprehensive medicinal chemistry reviews—they collectively underscore a common objective: the rational development of molecules capable of addressing unmet biomedical challenges.

The original research articles illustrate the continued evolution of synthetic organic chemistry as an enabling discipline for drug discovery. The development of novel C2-functionalized adenine nucleosides with reactive β-iodovinylsulfone and β-ketosulfone motifs expands the chemical toolbox available for nucleic acid modification and polymerase-mediated DNA engineering, highlighting opportunities for future applications in chemical biology and nucleic acid therapeutics [Contribution 1].

At the same time, the synthesis and biological evaluation of new aza-acridine derivatives demonstrate how careful scaffold optimization, combined with stability studies and computational analyses, can improve the pharmacological potential of established anticancer chemotypes while providing valuable structure–stability–activity relationships [Contribution 2].

Equally noteworthy is the elegant total synthesis of 22-hydroxyacuminatine, norketoyobyrine, and naucleficine, which showcases the enduring importance of synthetic innovation in accessing structurally complex natural products. Beyond the successful preparation of these challenging alkaloids, the work establishes versatile synthetic strategies that will facilitate future structure–activity relationship studies and the generation of novel analogs with improved biological properties [Contribution 3].

Complementing these original contributions, the comprehensive reviews reinforce the central role of medicinal chemistry in integrating chemical synthesis with biological function. The review on hydrazides highlights the remarkable versatility of this functional group as both a synthetic intermediate and a privileged pharmacophore, providing an up-to-date resource that will undoubtedly guide future efforts in designing biologically active molecules across multiple therapeutic areas [Contribution 4].

Similarly, the overview of *N*-alkyl deoxynojirimycin derivatives offers an insightful assessment of host-directed antiviral strategies, emphasizing the growing importance of targeting host cellular pathways to combat emerging viral diseases while minimizing resistance development [Contribution 5].

Taken together, these manuscripts illustrate several defining trends in modern medicinal chemistry. First, successful drug discovery increasingly relies on interdisciplinary integration, where synthetic chemistry, computational approaches, biological evaluation, mechanistic studies, and molecular pharmacology converge to accelerate the identification of promising therapeutic candidates. Second, advances in synthetic methodology continue to unlock previously inaccessible chemical space, enabling the preparation of increasingly sophisticated molecular architectures with tailored biological functions. Finally, the growing emphasis on mechanistic understanding, molecular stability, and rational optimization reflects the maturation of the field toward more predictive and translational research.

As Guest Editors, we are particularly encouraged by the diversity of scientific approaches represented in this collection. The studies reported herein not only advance fundamental knowledge but also provide valuable methodologies, molecular frameworks, and conceptual insights that are likely to stimulate future investigations across medicinal chemistry, chemical biology, pharmaceutical sciences, and drug development. The convergence of innovative synthetic strategies with rigorous biological validation exemplifies the multidisciplinary spirit required to address today’s most pressing biomedical challenges.

We sincerely thank all authors for their valuable scientific contributions, the reviewers for their thoughtful evaluations and constructive recommendations, and the editorial staff of *Molecules* for their professionalism and continuous support throughout the editorial process. We hope that the work presented in this collection will inspire new collaborations, foster innovative research directions, and ultimately contribute to the discovery of safer and more effective therapeutic agents for the benefit of human health.

